# Supplementary material for: Presence of a home cage running wheel, but not wheel running per se, decreases social motivation in adult C57BL/6J female mice
Source: PLoS One. 2026 Jul 28;21(7):e0353843. doi: 10.1371/journal.pone.0353843 (PMC13412059; doi:10.1371/journal.pone.0353843)
Supplement: Attachment — Submitted filename: Response to Reviewers.pdf [file pone.0353843.s001.pdf]

We thank the Reviewers and the Editor for their thoughtful and helpful comments. We believe that our manuscript has improved after addressing these concerns. Below, we address in turn the specific concerns raised by each Reviewer.

Reviewer 1

(1a) The experiments involve many confounding factors, which make difficult to interpret the results. First, control and experimental cages are markedly different in terms of shelter, running wheels, their colors, transparency, shapes and size of items, and free space. Particularly, the shelter in control cages would have considerable effect on sociality and anxiety, because it provides dark space with opaque walls. It is possible that the sociality in control mice was enhanced by the shelter, rather than decreasing effect of running wheels in experimental mice. If authors want to assess the effect of access to running wheels, fixed (no moving) running wheels should be placed in the control cages.

We agree with the Reviewer and thank them for this insightful suggestion. We have added a control experiment to test whether running behavior per se accounts for the effects of home cage running wheels on the social behaviors of female mice (Experiment 3). In this new experiment, we found that running per se cannot account for the inhibitory effects of home cage running wheels on female social behavior, because females raised with “immobile” running wheels exhibited low levels of social investigation and total USVs that did not differ from females raised with “mobile” running wheels (new Figure 6). We have changed the title of the manuscript and have edited each of the manuscript sections to reflect these new data and the altered interpretation of our findings.

(1b) Second, one running wheel to group-housing mice would induce competition that causes chronic stress. By the competition, the setting may not “free access” situation.

We agree with the Reviewer, and the inclusion of the new immobile wheel control experiment (Experiment 3) rules out the possibility that either running per se or competition for running time can account for the inhibitory effects of home cage running wheels on female mouse social behavior.

(2a) The behavioral analysis methods appear to be too rough. Social interaction contains multiple aspect of behavior, which needs to be analyzed by multiple tests, such as three-chamber tests, social preference/novelty tests, and animal tracking in their interactions in home cages. Authors need to examine not only the interaction with strangers, but also with familiar mice.

Much of our lab’s work focuses on the behavioral contexts and neural circuits that promote USV production in female mice. Although we have never published the observation, we have observed that females produce higher rates of USVs during free social interactions than during three-chamber preference tests. We have also observed consistently that female mice exhibit lower rates of both social investigation and USV production during interactions with familiar females compared to interactions with novel females. Thus, to evaluate the effects of home cage running wheels on both female social investigation and female-female USV production, we focused our experiments on quantifying social investigation and total USVs during free social

interactions between subject females and novel females. We have elaborated on this rationale within the Results section (lines 64 and 68-71). Although we agree with the Reviewer that it could be of interest to evaluate the effects of home cage running wheels on female social behaviors in additional behavioral assays, the inclusion of such assays goes beyond the aims of the current study.

(2b) Besides, in Experiment 2b and c, mice were tested in the social interaction test repeatedly. Even though stranger mice were different, the experimental mice would be habituated to the test, and the second results may not be accurate. Authors need to confirm the results using the mice that experience the test for the first time.

In Experiments 2b and 2c, subject females were given two social interaction tests, separated by a period of 2 weeks, each with a different novel female. To our knowledge, there is no literature to suggest that the social behaviors of female mice systematically differ as a function of testing order in free social interactions separated by an interval of weeks. If anything, habituation to the testing chamber and testing procedure might be expected to increase, not decrease, female social investigation. Moreover, both groups of females (those raised with huts and those raised with wheels) were given a second social interaction test following the 2-week period of enrichment change (hut-to-wheel or wheel-to-hut). If the decrease in social investigation in the hut-to-wheel females were due to a more general order effect of the social interaction tests, we would have expected to also see a decrease in social investigation time in the wheel-to-hut females. The lack of such an effect indicates that the change in female social investigation time is driven by the 2-week period with the home cage running wheel, rather than by an order effect of the social interaction tests.

Reviewer 2

(1) While the manuscript is generally clear, the specific number of animals used per experimental group is not explicitly stated in the Materials and Methods section. The sample sizes are mentioned for some analyses within the Results section (e.g., "n = 22" in Line 209, "n = 20" in Line 233), but providing a comprehensive summary of the N for each group (Hut and Wheel) in each experiment (Exp 1, 2a, 2b, 2c) within the Methods section would enhance transparency. This would help readers to quickly assess the statistical power and scale of the study.

We have added this information to the Methods section, at the end of the Subjects sub-section (lines 103-106).

(2) The authors specify a housing density of "2-3 siblings" per cage for Experiment 2. However, the number of mice housed per cage in Experiment 1 is not mentioned. As housing density is a critical variable that can influence social dynamics and behavior, clarifying this detail for Experiment 1 is important. Providing this information would ensure that the housing conditions are comparable across experiments and would strengthen the replicability of the findings.

We have added this information to the Methods (lines 113-114).

3. The description of the social interaction test (Lines 107-116) is detailed, but it omits the duration of the habituation period. Specifically, it is unclear how long the subject mouse was left in its home cage within the sound-attenuating chamber before the novel female was introduced. The transfer to the recording chamber and the removal of cage mates are potentially stressful events. Specifying the length of this habituation period (or its absence) is crucial, as it could influence the subject's anxiety levels and subsequent social behaviors.

We did not use a habituation period, and we have added this information to the Methods (lines 125-126).
