# Supplementary material for: Presence of a home cage running wheel, but not wheel running per se, decreases social motivation in adult C57BL/6J female mice
Source: PLoS One. 2026 Jul 28;21(7):e0353843. doi: 10.1371/journal.pone.0353843 (PMC13412059; doi:10.1371/journal.pone.0353843)
Supplement: Attachment — Submitted filename: Response_to_Reviewers_auresp_1.pdf [file pone.0353843.s002.pdf]

We thank the Reviewer and the Editor for their thoughtful and helpful comments. We believe that our manuscript has improved after addressing these concerns. Below, we address in turn the specific concerns raised.

Academic Editor

1. *Please ensure that your manuscript meets PLOS ONE's style requirements, including those for file naming.*

We have formatted the manuscript according to PLOS ONE's style requirements, although these formatting changes are not marked by highlighting in the revised manuscript.

2. *To comply with PLOS One submissions requirements, in your Methods section, please provide additional information regarding the experiments involving animals and ensure you have included details on (1) methods of sacrifice, (2) methods of anesthesia and/or analgesia, and (3) efforts to alleviate suffering.*

We have added information to the Methods about how animals were either (1) euthanized or (2) used as breeders at the end of behavioral experiments. The behavioral experiments themselves did not cause suffering, and we have also added a statement to that effect (lines 95-107).

3. *In the online submission form, you indicated that your data will be submitted to a repository upon acceptance. We strongly recommend all authors deposit their data before acceptance, as the process can be lengthy and hold up publication timelines. Please note that, though access restrictions are acceptable now, your entire minimal dataset will need to be made freely accessible if your manuscript is accepted for publication. This policy applies to all data except where public deposition would breach compliance with the protocol approved by your research ethics board. If you are unable to adhere to our open data policy, please kindly revise your statement to explain your reasoning and we will seek the editor's input on an exemption.*

We are working to deposit our data now, and the entire minimal dataset will be freely accessible upon acceptance as follows:

Patryk Ziobro, Cassidy Malone, Stephen Batter, Longao Xu , Selina Xu, Alina Logonov, Katherine Tschida. (2026) Data from: Presence of a home cage running wheel, but not wheel running per se, decreases social motivation in adult C57BL/6J female mice. [Data set] Cornell University Library eCommons Repository. https://doi.org/10.7298/4b87-fh59

Please note that this is a placeholder DOI, and the link will resolve once we have finalized our data deposit (likely within a week or two).

4. *If the reviewer comments include a recommendation to cite specific previously published works, please review and evaluate these publications to determine whether they are relevant and should be cited. There is no requirement to cite these works unless the editor has indicated otherwise.*

Noted.

5. *Please review your reference list to ensure that it is complete and correct. If you have cited papers that have been retracted, please include the rationale for doing so in the manuscript text, or remove these references and replace them with relevant current references. Any changes to the reference list should be mentioned in the rebuttal letter that accompanies your revised manuscript. If you need to cite a retracted article, indicate the article's retracted status in the References list and also include a citation and full reference for the retraction notice.*

No publications we have cited have been retracted.

6. *Additional Editor Comments (if provided): One of the study's main findings is that the mere presence of a running wheel in the home cage during adolescence, rather than wheel running per se, alters the subjects' adult social behavior. In addition to the explanations provided by the Reviewers and addressed in the Discussion, another possibility is that the size of the wheel itself affects the mice. Unlike the paper hut, which in itself (with its thin walls and no base) occupies little space in the home cage and may even provide a sheltered area in which mice can huddle together and socialize, the running wheel takes up a substantial portion of the available cage space. This reduced space may limit opportunities for social play and huddling. As Reviewer 1 suggested, the wheel may also act as a stressor or promote competition among cage mates. In this way, mice may grow up during adolescence with fewer opportunities for social interaction. The results of Experiment 3 are consistent with this possibility. An appropriate control condition would be a home cage containing an object other than a running wheel but matched in size, or alternatively a smaller home cage.*

Thank you for these useful suggestions- we have added mention of these additional possibilities to our Discussion (lines 353-357).

This study was started by an undergraduate honors student who graduated 2 years ago and by a graduate student who is defending this summer. Given these timelines, we are not planning to pursue additional control experiments to distinguish between these possibilities.

Reviewer 1

1. *Minor revision- The paper would benefit from additional primary literature to support claims of increased anxiety and differences in nesting behavior with the presence of a wheel or other forms of environmental enrichment.*

As mentioned in the Introduction, there is a rich literature on the effects of running wheels (and other forms of enrichment) on ameliorating the effects of various experimenter-introduced stressors. We are not aware of other studies that have evaluated the effects of home cage running wheels on anxiety, nesting behavior, or social behaviors in otherwise unstressed rodents.

Past work has shown that rodents prefer cages with nesting material over those without (Van De Weerd et al., 1998) and that the absence of nesting material increases anxiety-like behaviors in

B6 mice (Kulesskaya et al., 2011). Because cages with running wheels did not have paper huts, these cages then also had less available nesting material, which could in turn have impacted anxiety and/or social behaviors in females. We have added these references to the Discussion (lines 343-348).

2. *Minor revision- While the rationale for using females in the study is stated (lines 63-66), additional context of sex differences in behavior would strengthen the argument for using females.*

We have also modified the Introduction (line 67), as well as the last sentence of the Discussion (371-375) to help clarify the sex differences in behavior that underlie our rationale.

3. *Minor revision- Suggestion for clarity, to be consistent with the descriptions of the experiments in the rest of the paper. Add the phrase "experiment 3" to the text (line 81).*

We have made this change- thank you.
